# MAP3D: An explorative approach for automatic mapping of real-world eye-tracking data on a virtual 3D model

**DOI:** 10.16910/jemr.15.3.8

**Published:** 2023-05-31

**Authors:** Isabell Stein, Helen Jossberger, Hans Gruber

**Affiliations:** University of Regensburg, Germany; University of Turku, Finland

**Keywords:** 3D stimuli, automatic fixation mapping, eye movement, eye tracking, photogrammetry, virtual 3D model

## Abstract

Mobile eye tracking helps to investigate real-world settings, in which participants can move freely. This enhances the studies’ ecological validity but poses challenges for the analysis. Often, the 3D stimulus is reduced to a 2D image (reference view) and the fixations are manually mapped to this 2D image. This leads to a loss of information about the three-dimensionality of the stimulus. Using several reference images, from different perspectives, poses new problems, in particular concerning the mapping of fixations in the transition areas between two reference views. A newly developed approach (MAP3D) is presented that enables generating a 3D model and automatic mapping of fixations to this virtual 3D model of the stimulus. This avoids problems with the reduction to a 2D reference image and with transitions between images. The x, y and z coordinates of the fixations are available as a point cloud and as .csv output. First exploratory application and evaluation tests are promising: MAP3D offers innovative ways of post-hoc mapping fixation data on 3D stimuli with open-source software and thus provides cost-efficient new avenues for research.

## Introduction

**Figure 1.**
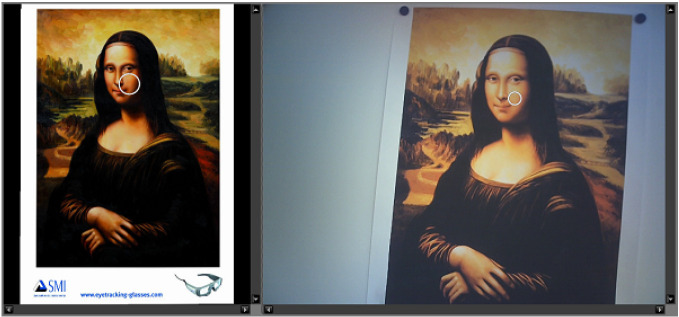
Gaze mapping in BeGaze. The left side shows the reference view and the right side shows one frame of the video of the scene camera with the fixation visualized as a circle. Source: SMI BeGaze Manual.

Eye movement research has been restricted to laboratory settings for a long time. The technology of mobile eye tracking allows doing research in real-life settings. Using mobile eye-tracking data on stimuli in the real, three-dimensional world offers many opportunities for research in various domains. One potential advantage of mobile eye tracking is that ecological validity is high, as the data collection can take place in real-world settings, in which participants move freely. According to Lappi, “modelling human cognition and behavior in rich naturalistic settings and under conditions of free movement of the head and body – ‘in the wild’ – is a major goal of visual science” (13, p. 1). However, analyzing eye-tracking data from the real world is challenging. What usually happens, while analyzing the data of a video-based mobile eye tracker is that the 3D stimulus is reduced to a 2D photo or filmstill from the eye-tracking video to map the fixations from the video on this static 2D reference view. This happens frame by frame or as a so-called semantic gaze mapping, which means mapping fixation by fixation on the static reference image. “This procedure is necessary to analyze aggregated experiment results.” (2, p. 1). To find out what a participant is looking at and being able to compare the gaze data of several different participants, a common reference, on which every single fixation can be mapped, is required (2). An example of the mapping process is shown in Figure 1.

The reduction to a 2D reference image during this kind of mapping leads to a loss of information about the three-dimensionality of the stimulus as only one view of the stimulus from a certain angle is included in the analysis. One possible solution to cope with this problem is working with multiple reference views to analyze the various perspectives during the viewing process. However, this procedure also leads to a lack of information when switching from one reference view to another. Moreover, in some cases, it is difficult deciding on which reference view a certain fixation has to be mapped (cf. 25). Analyzing the gaze movements is also more challenging because the calculations have to be carried out over all reference views for each participant.

Many studies deal with the examination of 3D stimuli in real life, but the three-dimensionality of real-world objects is usually not taken into account. For some research questions, three-dimensionality indeed can be neglected. For example, in consumer research (5), the goal might be answering a question like: "Has the participant fixated the product on the shelf while shopping in the supermarket?" In this case, the evaluation via a 2D reference image with 2D areas of interest (AOI) is sufficient. Moreover, research questions relating to the raw data of the eye tracker (e.g.: "How many fixations did the subject perform in a given time?") can be answered without the need for a common reference, no matter if it is 2D or 3D. In some research areas, however, information about the 3D nature of the stimulus is relevant. For example, Wang et al. (27) investigated human perception in 3D with a remote eye tracker in their study. They provided a large dataset of human fixations on real-world 3D objects presented in different viewing conditions. Here, the three-dimensionality is relevant, and the focus of the study. Mobile eye tracking is also used in 3D research. For example, Stein et al. (25) investigated the perception of artists while looking at 3D artwork. Although many studies take place in the real world, only a few researchers have addressed the 3D to 2D conversion problem. One reason for the research gap is the difficult technical feasibility because current eye-tracking software solutions for mobile eye trackers do not offer a common 3D reference to map the fixations on it. In the following, research approaches are presented which have dealt with a 3D reference.

### Analyzing with a 3D Reference

To be able to analyze gaze data on a 3D reference it is necessary to generate a virtual 3D model of the stimulus. There are different ways to generate this kind of 3D reference. Pfeiffer and Renner (19) and Pfeiffer et al. (20) developed the EyeSee3D method, a model-based analysis of mobile eye tracking in static and dynamic 3D scenes. The gaze rays will be computed automatically and in real-time. The method uses markers (fiducial augmented reality markers) for the automatic mapping (also called coding) on the virtual 3D model. The virtual model is an abstract model of the environment with geometric shapes around the figures (stimuli). These geometric shapes serve as an abstract model and they represent a 3D area of interest (AOI) around a 3D stimulus (20). The use of such 3D areas of interest is sufficient for some particular research questions. For example, it is possible to count the fixations on certain objects, or it is possible to investigate in which order different objects were fixated. However, it is not possible to identify which area on the object was fixated. It is either a hit on the target or not. Wang et al. (28) also used markers. Their virtual model is detailed and fits the geometry of the stimulus accurately. They use a 3D print of the virtual model for their stimuli. The high accuracy of this system is based on the fact that participants are not allowed to move around during data collection. In addition, the distance between the participant and the stimulus has to remain stable during the recording. As a consequence, this setting is quite different from doing research “in the wild” as stated by Lappi (13). For many research questions in the real world, it is necessary that participants have the opportunity to move freely in the environment (13). Moving freely is, for example, required when investigating perception in an art museum where participants explore an exhibition. Another limitation of the approach of Wang et al. (28) is that the markers have to be included in the 3D-printed stimulus, which sometimes is difficult or even impossible. In some environments, it is impossible to place markers on or even near the stimulus. For example, in an art museum, it is usually not allowed to fixate markers on artwork. Moreover, markers in the field of view can affect the eye movements of the participants.

In the following, we explore an alternative to create an accurate 3D reference of the stimulus without the need for distracting markers, without the expensive equipment for 3D scanning, and with the opportunity for the participants to move freely in the environment. The technique we used to design a virtual 3D model and introduce here is called photogrammetry.

### Photo- and Videogrammetry

Photogrammetry is a technique for creating virtual 3D models by using photos from different perspectives on the stimulus. Photogrammetry is used in many professional domains. Application examples are architecture and cultural heritage, engineering surveying and civil engineering, industrial applications, forensic applications and medicine (14). Videogrammetry is based on the technical principles of photogrammetry and computer vision and uses video components such as video cameras for image acquisition (6). Already in 1997, Gruen emphasized the potential of this technology. “Videogrammetry is per se fully 3D, it works in a non-contact mode, determines and tracks even very complex point clouds with a high number of particles, delivers very precise and reliable results, and can be fully automated.” (6, p. 156). According to Remondino (21), photogrammetry and videogrammetry derive precise and reliable 3D metric information from multiple images. For the traditional photogrammetry methods, the 3D location and pose of the camera or the 3D location of ground control are required. A more recent method, called Structure from Motion (SfM), solves the camera pose and scene geometry simultaneously and automatically uses a highly redundant bundle adjustment based on matching features in multiple overlapping offset images (30). Photogrammetry works best without highly reflective objects and surfaces as these can disturb the photogrammetry algorithms (29).

### Photogrammetry and Eye Tracking

The combination of photogrammetry with eye tracking and the SfM technique has already been used by several researchers. For example, the developers of the Pupil Labs eye tracker described in their Master thesis conducted at the Massachusetts Institute of Technology (9) that the SfM technique is necessary for the final step of their software tool chain. The user can observe and analyze the subject’s patterns of visual attention as they move through space. The result is a 3D representation of the space by merging it into a single representation. “This representation reveals: The subject’s movements as a path through a space, his capture routine, a three-dimensional point cloud construction as calculated by the SfM pipeline, and the patterns of visual attention as three-dimensional projections.” (9, p. 134). They worked with SfM to be able to depict the subjective space of the subject’s perception. It was not intended to compare patterns of visual attention across several subjects on a common reference. Schöning et al. (22) also combined SfM with eye tracking. They presented a 3D reconstruction pipeline in their work that implements content awareness by combining a world camera of an eye tracker with gaze information. The goal of their work was to identify AOIs within the video sequences. Their field of research is the area of computer vision to develop new ways for assistive technologies and human-robot interaction (22). Jensen et al. (8) also used SfM. They tested their approach in a case study to create 3D AOIs and to do 3D mapping of visual attention on shelves in a supermarket. Jensen et al. (8) visualized their data with heat maps on the point clouds. Singh et al. (23) used photogrammetry to capture real-world gaze behavior on a 3D model of the environment. Singh et al.’s (23) work is based on heat maps and spotlights. These authors show a way to analyze eye-tracking data only for the Sensomotoric Instrument (SMI) glasses. The software they recommend for creating a 3D point cloud for the 3D model is commercial and therefore not freely available to researchers. Kollert et al. (12) used the technique of video- and photogrammetry in combination with eye tracking in their study, in which they recorded eye movements in urban outdoor environments. They created 3D heat maps by using a Tobii eye tracker and commercial software for the event detection and SfM technique. To improve their 3D model of the environment, they used a terrestrial laser scanner (LiDAR)enabling georeferencing.

### Aim of the Explorative Approach

To tackle the described challenges in mobile eye-tracking research, we propose an open-source solution for creating a 3D model of a stimulus and show how photogrammetry and SfM can be used for analyzing eye movements. The aim was to develop, explore and test an approach for the automatic mapping of real-world eye-tracking data on a virtual 3D model, which we called MAP3D. With the help of MAP3D, we aim at generating a virtual 3D model of a stimulus, without the need for markers and with the opportunity to move freely in the setting. The eye movements should be automatically mapped onto this virtual 3D model without the need for manual mapping allowing a more efficient work process for researchers. The x, y and z coordinates of the fixations should be available. It should also be possible to map fixations from multiple participants to the same 3D model, in the same coordinate system. If the fixations of all participants can be mapped to the same reference, it allows comparisons between the participants for later application in eye-tracking studies. At this stage, the focus was on the fixations and not on the whole gaze data. In the following, MAP3D is explained and its application is exploratively tested to evaluate accuracy and feasibility.

## MAP3D

To create a virtual 3D model of the stimulus without the need for additional technical equipment we used photogrammetry. As the fixation detection is performed by the eye-tracking software, MAP3D can use the provided information to map the fixations on a 3D model. Due to the technical conditions of MAP3D, all relevant information for automated coding is given. While the basic procedure is suitable for all common mobile eye trackers, MAP3D was initially based on the data structure of the Pupil Labs software as a starting point. In the following paragraphs, the workflow of MAP3D is presented.

### Creating a Virtual 3D Model of the Stimulus

To create a lifelike 3D model on which the fixations of participants are mapped to be able to compare these with this particular model or so-called common reference, the 3D model should meet the following criteria: accurate stimulus representation, rotation possibility, zoom function, visualization of the surface, and 3D visualization of the whole fixation sequence.

**Figure 2.**
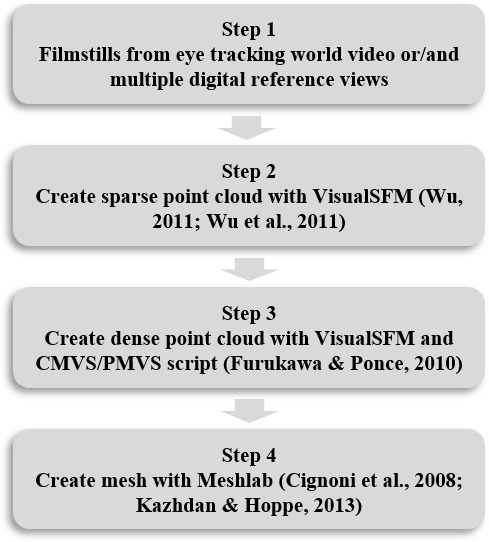
MAP3D workflow for creating a virtual 3D model.

There are several methods to create a virtual 3D model using photogrammetry. For further analysis of the eye movements, it was necessary to choose a method that not only creates a 3D model, but also preserves the camera data. The chosen method consists of four steps. These steps and the used open-source programs are shown in Figure 2.

**Figure 3.**
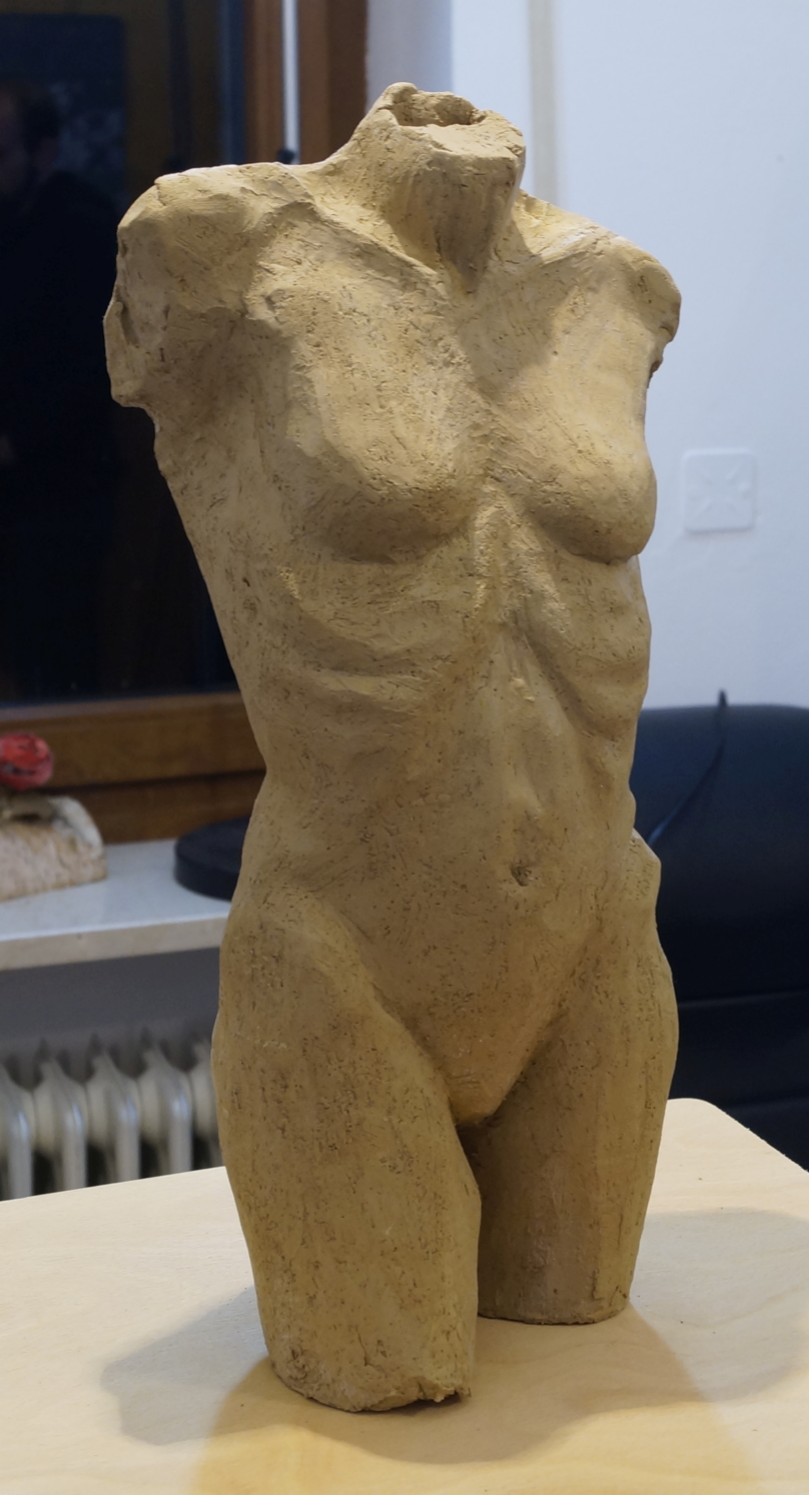
Example of a picture of the 3D artwork used to create the 3D model.

The *first step* of the workflow involves taking multiple pictures of the stimulus from different perspectives. The stimulus for our exploration was a clay-based artwork showing a female torso (26). 86 pictures of the torso (see an example in Figure 3) were used to create a virtual 3D model of the stimulus.

The pictures were taken with a digital camera. The total number of pictures is not as important as covering all the details of the stimulus from multiple perspectives. Alternatively, it is also possible to use the scene or world camera of the eye tracker for all pictures. The higher the resolution of the camera, the better the accuracy and feature detection of the photogrammetry. Poor camera resolution or the use of photos with very different resolutions can lead to problems with the reconstruction. Also strongly changing lighting conditions, in the reference images or the images from the eye-tracking video, can influence feature detection.

**Figure 4.**
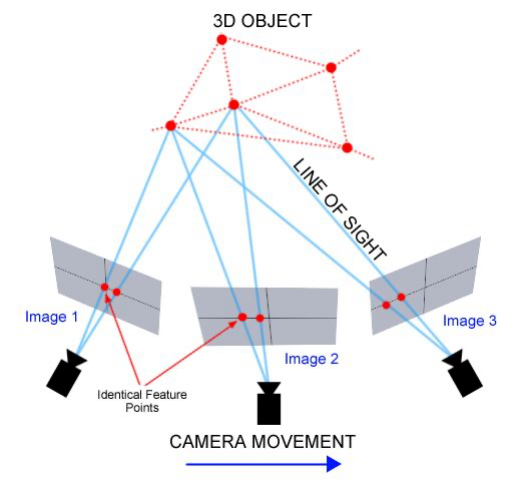
Photogrammetry basics. © Mason, A. (15). Making 3D models with photogrammetry. The Haskins Society.

In the *second step* of the workflow, the multiple images are loaded into the open-source program VisualSFM (31; 32). The SfM technique, which is the basis of VisualSFM, computes a sparse point cloud of the stimulus. In other words, this means that identical feature points in the different images are identified and these feature matches are computed in VisualSFM. These identical feature points are needed to calculate the relationship between the different origins and perspectives of the pictures (see Figure 4).

Furthermore, VisualSFM reconstructs the position, orientation (relative to the reconstruction) and focal length of the camera of each (input) image. This camera data is also necessary for the later projection of the fixations onto the 3D model.

After identical feature points are identified, the function ‘Compute 3D Reconstruction’ or ‘Reconstruct Sparse’ can be started to receive a set of data points (the feature points) in a 3D coordinate system. This set of points is called a sparse point cloud.

**Figure 5.**
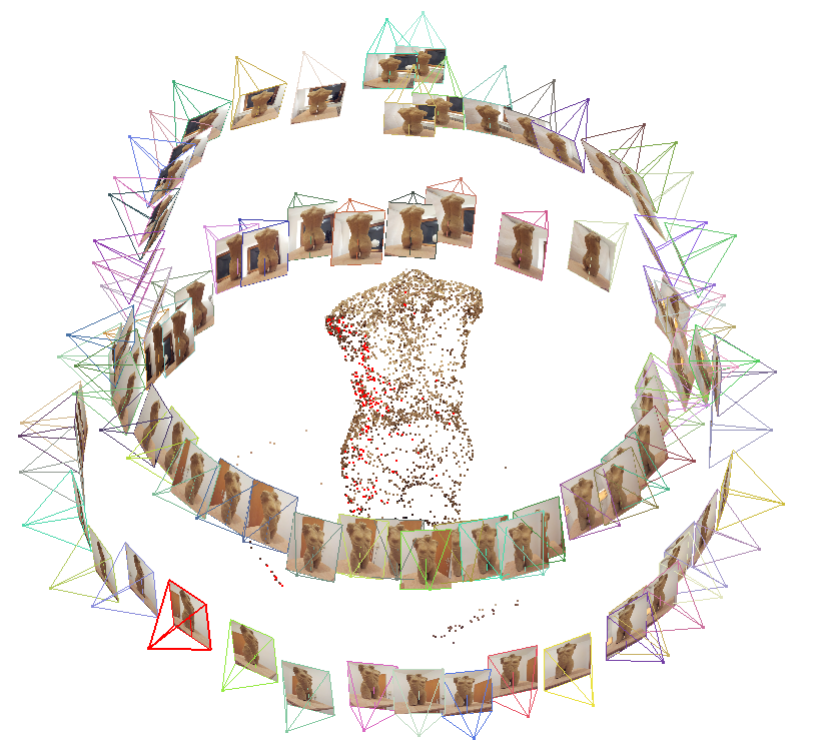
Screenshot of the sparse point cloud in VisualSFM with the picture sources around the stimulus.

Figure 5 shows such a sparse point cloud and displays that VisualSFM computes the different origins of the pictures or frames, which were loaded into the program. One can see the origins of the 86 pictures of the digital camera in two circles around the stimulus.

**Figure 6.**
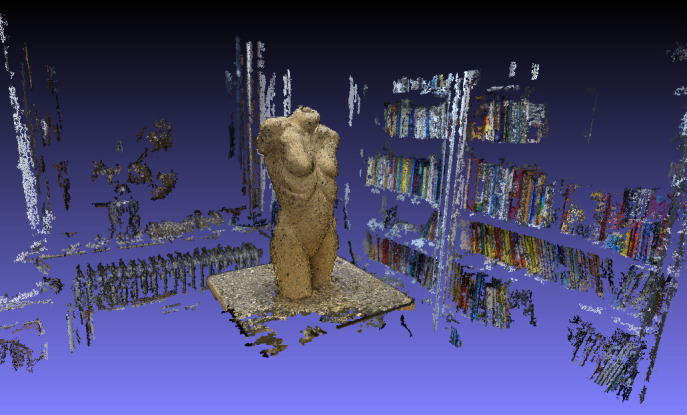
Screenshot of the dense point cloud in Meshlab with the surrounding of the setting.

In the *third step* of the workflow, a dense point cloud is created. To realize the dense point cloud, the CMVS-PMVS script (4), which can be integrated into the VisualSFM software, is used by applying clustered and/or patched-based multi-view stereo (CMVS/PMVS) algorithms. The function CMVS has to be run in the VisualSFM program to receive a more detailed and denser point cloud. This step can take a while corresponding to the number of pictures loaded. For *step four* of the workflow, the dense point cloud has to be imported into the program Meshlab (see Figure 6).

**Figure 7.**
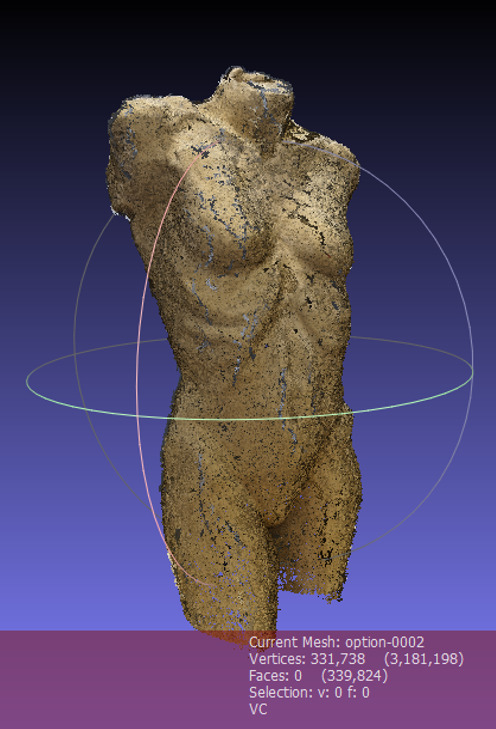
Screenshot of the dense point cloud of the 3D artwork in Meshlab.

In our following exploratory analyses, we were interested in the fixations on the stimulus and not the environment. Thus, the points not needed (whitespace) were deleted. Fixations on the environment can of course be included in the analysis if the research question requires it. The result of the cleaned dense point cloud of the stimulus is shown in Figure 7.

**Figure 8.**
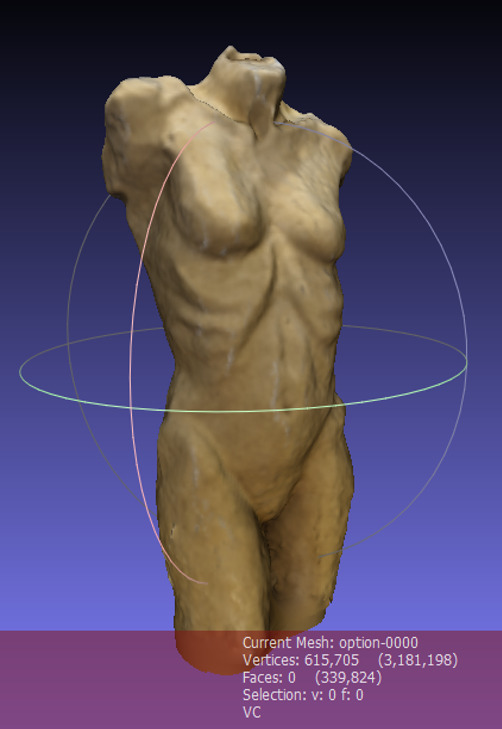
Meshed 3D model of the 3D artwork in Meshlab.

In Meshlab, the user can generate a virtual 3D model with a textured surface using the so-called Poisson surface reconstruction (11). The point cloud is computed to a mesh with vertices, edges and faces. The result is a complete copy of the 3D stimulus, which even has the surface structure and texture of the original stimulus. It is also possible to display the mesh without the texture in Meshlab, depending on the analysis the researcher addresses. In Figure 8, the 3D model of the stimulus with a textured surface is displayed.

### Transfer Eye-Tracking Data on the Virtual 3D Model

**Figure 9.**
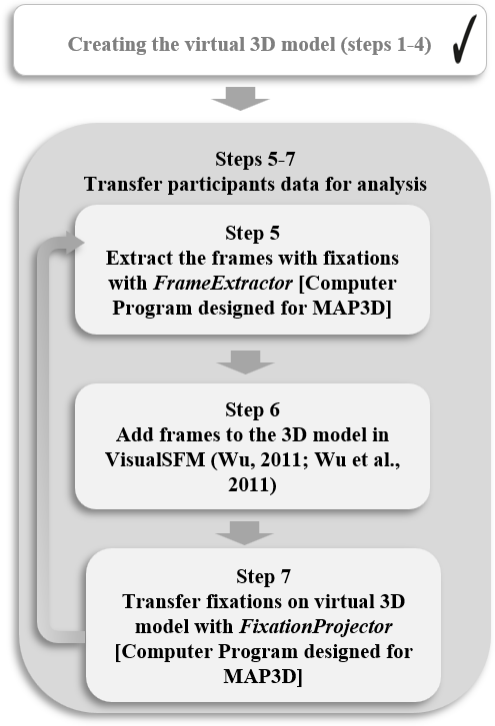
MAP3D workflow for transferring fixations on the virtual 3D model.

To be able to analyze the eye movements on the created virtual 3D model, the fixations of each participant have to be transferred to the 3D model. Therefore, the fixation frames have to be extracted (step 5), added to the 3D model (step 6) and projected to the 3D model (step 7) for each participant (see Figure 9).

To perform steps 5-7, command line programs were developed for the MAP3D approach by (7). The tools are the FrameExtractor and the FixationProjector. Later in the process, the FixationMarker is a useful tool for evaluation purposes.

For the *fifth step*, the so-called FrameExtractor was developed to extract the frames from the world video, in which the subject made a fixation. For each fixation the Pupil Labs eye-tracking software (10) has detected and saved in a fixation.csv file, the corresponding frame of the world video is extracted. In addition, the FrameExtractor writes a .csv file, which assigns fixations to images for further analysis. The FrameExtractor is programmed with C#. For participant 1, for example, there were 14 fixations and therefore, 14 frames were extracted.

**Figure 10.**
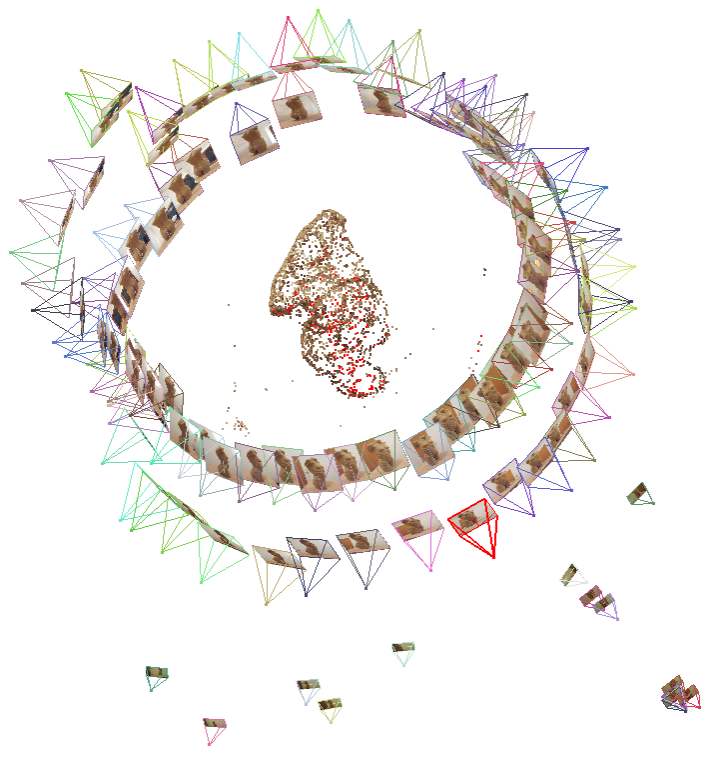
Screenshot of the sparse point cloud in VisualSFM with the picture sources around the stimulus and the frames with a fixation of participant 1.

In the *sixth step*, the VisualSFM project has to be opened. The extracted frames are added to the picture repository. As explained in the second step, feature matches need to be calculated for the new frames and the sparse point cloud reconstruction needs to be repeated to include the frames in the virtual space of the 3D model. Figure 10 depicts the 14 origins of the frames with a fixation from participant 1, who was standing outside of the circles with the reference images. Five of them are close together in the lower right corner. The movement and the distance of the participant to the stimulus become clear.

**Figure 11.**
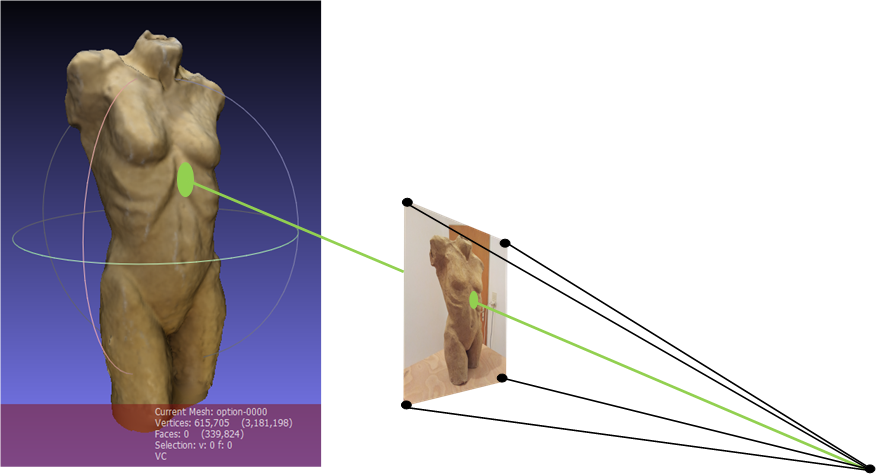
Transferring fixation data on the 3D model.

To perform the *seventh step*, it is important to recall that photogrammetry detects the origin of every single picture used for creating the 3D model. The frames are taken from the scene camera of the eye tracker. The eye tracker also provides the subject’s angle of view in relation to the scene camera’s image. Combining the original location of the frame, which corresponds to the participant’s point of view, with the viewing angle, the location of the fixation can be determined. This is done by determining the intersection point of the straight line (visual beam) with the surface of the 3D model (see Figure 11).

The transfer of the fixations to the model can be done manually, like the manual mapping or coding process from other eye-tracking software. However, a procedure was developed to automate this process, which otherwise would be time-consuming. The FixationProjector tool calculates where the visual beam collides with the 3D model and visualizes this in MeshLab. The FrameExtractor.csv output file is needed because here all fixations, corresponding pictures, origins, and fixation durations are listed.

**Figure 12.**
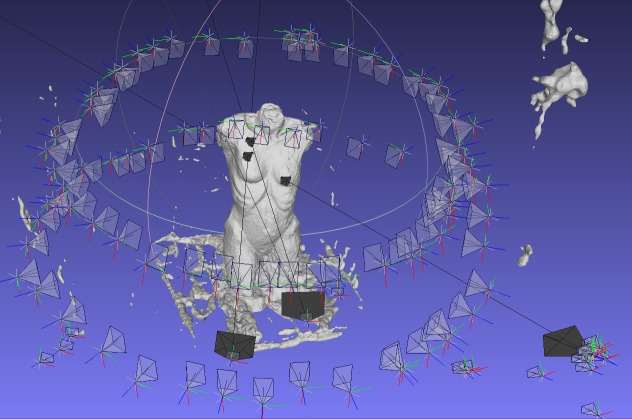
Transferring fixation data with the FixationProjector tool in Meshlab.

The coordinates of the intersection points are additionally written in a .csv file. These 3D fixations are available as a point cloud in a .ply file. Additionally, a MeshLab project file is created containing the 3D model as well as all fixations of the participant and all so-called raster cameras, meaning all cameras used by photogrammetry. Figure 12 displays how the FixationProjector calculates three fixations hitting the 3D model.

## Application Test of the MAP3D Work-flow: A Demonstration

### Preparation

To try out the newly developed MAP3D approach, the eye movements of two participants were tracked. Both participants had normal vision. The eye movements were recorded with the 120Hz mobile Pupil Labs eye tracker with an average gaze estimation accuracy of 0.6 degrees of visual angle and 0.08 degrees of precision. Precision is calculated as the Root Mean Square (RMS) (10). The eye tracker has a 60Hz high-speed 2D world camera with a 60 degree FOV (field of view) lens.

The stimulus (see Figure 3) was a female nude sculpture (height: 36 cm). The eye tracker was calibrated with the so-called “manual marker calibration” from Pupil Labs. The 3D artwork was displayed on a modelling trestle in the middle of a room, with enough space to walk around the stimulus. The participants had the task to explore the artwork freely and for as long as they wanted. No instruction for the direction of walking was given. The lighting conditions were the same for both participants.

The reference views for the photogrammetry were taken with a digital camera, at two different heights around the stimulus. Pupil Player was used to process the data and perform fixation detection. A fixation had a maximum dispersion of 3° and a minimum duration of 30 ms.

A 3D model was created according to steps 1-4 of the MAP3D workflow. The whole fixation sequence was summarized in one virtual 3D model. The fixations were transferred automatically to the model according to steps 5-7 of the MAP3D workflow.

### Mapping Process and Provided Data

All fixations are included in one common 3D coordinate system with x, y and z coordinates. The previously video-based 2D data can now be assigned to an object in the 3D space.

**Table 1.**
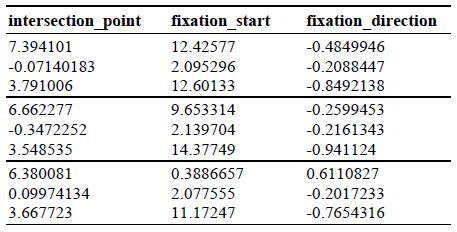
Example of 3D coordinates from the csv. output.

For quantitative analysis of the fixation data, the .csv file and the .ply file with the point cloud of the 3D fixations created by the FixationProjector can be used. Table 1 shows a snippet of the .csv file. The user receives the x, y, and z coordinates of the “intersection point”, where the visual beam hits the 3D model of the stimulus. The “intersection point” can also be called the fixation point. In the second column, the “fixation start” is listed. The fixation start is the origin of the visual beam which has been determined by the photogrammetry algorithm. In other words, this is the position of the world camera at the time the fixation was detected. Additionally, these points are also described in the 3D space with x, y and z coordinates. In the third column, the “fixation direction” is presented, which describes the direction of the visual beam as a 3D vector. If there is no intersection between the vector and the 3D model, no intersection point is calculated. In an extra column named “status”, this case is described as “no intersection between fixation vector and model” in the .csv file meaning that the fixation was in the whitespace.

In the .csv file, each line contains the data of one fixation. The order of the fixations in the file is identical to the order in which the fixations were made. Additionally to the data shown in Table 1, the .csv file contains the data of the eye-tracking software for each fixation (e.g. fixation duration, timestamp, pupil size).

For some fixations, no automatic mapping was performed during the first test. Apparently, the corresponding frames did not match during the creation of the sparse point cloud. For these cases, it was possible to identify the correspondingly marked fixations from the .csv file and to map them manually. Even though not all of the fixations were automatically transferred to the 3D model, the first results of the application test were promising. Therefore, additional tests were performed to verify and evaluate the process in more detail.

### Evaluating MAP3D

To evaluate the MAP3D tools, three different tests were performed. The first evaluation test verified whether the correct positions of fixations are identified in the different steps of MAP3D. In this test, the following criteria to be achieved were set: The fixations need to be correctly registered and mapped in all steps.

The second test aimed at evaluating the complete procedure of the MAP3D approach by adding red dots to the stimulus checking fixation detection and automatic mapping. In this test, the following criteria to be achieved were set: All fixations made are mapped to the 3D model and all red dots are hit.

Finally, in the third evaluation test, the accuracy of the automatic and manual mapping is compared. In this test, the following criterion to be achieved was set: The automatic mapping is more accurate than the manual mapping.

#### Evaluation Test I: Positions of Fixations in Each Step of the Workflow

**Figure 13.**
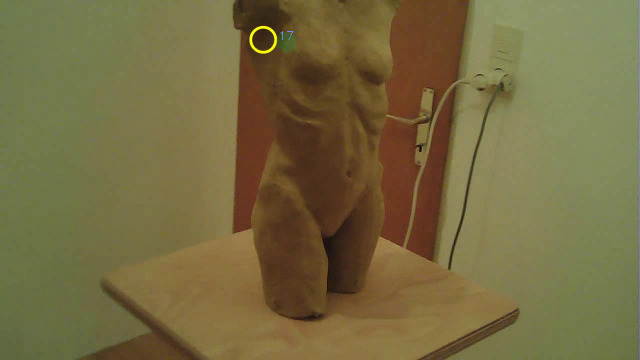
Filmstill of the frame which shows fixation (ID 17) in Pupil Labs world_viz_video.

To check the functionality of the MAP3D tools, the positions of the fixations were checked for their apparent correctness based on face validity. In order to demonstrate this functionality test, we use the fixation indicated with ID 17 as an example. The output of Pupil Labs is used as a starting point for the evaluation. Figure 13 shows a still image from the world_viz_video from Pupil Labs with the fixation. The figure shows the visualization of the fixation as captured by the fixation detection of Pupil Capture, detected and visualized with Pupil Player. The fixation with ID 17 is marked with a yellow circle.

**Figure 14.**
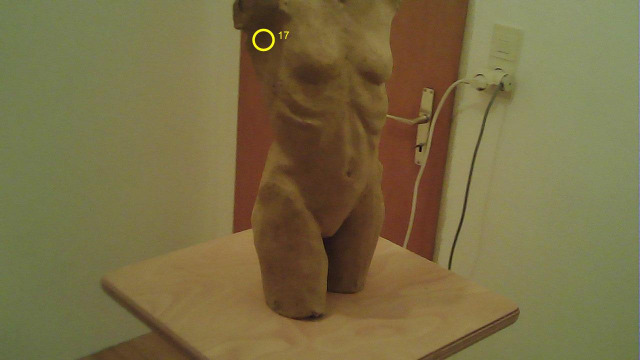
Image from a filmstill from the Pupil Labs world_video automatically generated by the MAP3D FixationMarker tool also showing fixation ID 17.

To visualize the output of the FrameExtractor, the MAP3D FixationMarker was created as an evaluation tool. Using the x and y coordinates from the fixation.csv file obtained from the Pupil Labs software, the FixationMarker marks the corresponding fixation in all images extracted by the FrameExtractor. The fixation is visualized with a yellow circle and the corresponding fixation ID (see Figure 14). Now, the yellow circle from the FixationMarker in Figure 14 can be compared with the yellow circle of the fixation point output from Pupil Labs shown in Figure 13. The two circles are congruent in the same place on the torso.

**Figure 15.**
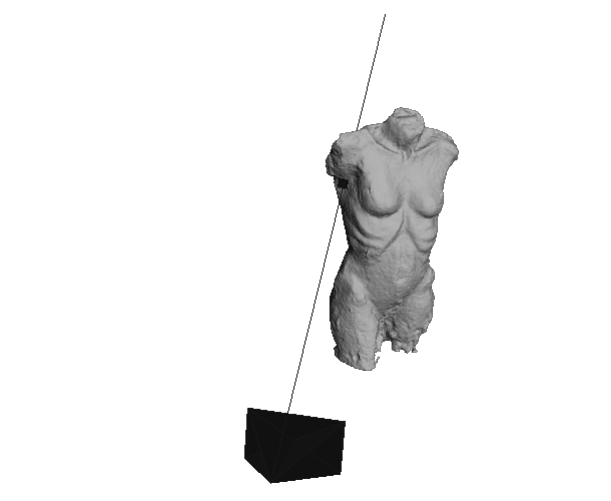
Image of the 3D model in MeshLab showing the camera origin of the world camera, the visual beam and the mapped fixation (ID 17) created with FixationProjector.

Furthermore, the positions of the fixations were checked with MeshLab in the 3D model. Therefore, the FixationProjector also provided a MeshLab project file containing the 3D model, the origins of the observer, the visual beams and the intersection points. An example of the fixation with ID 17 is shown in Figure 15. As depicted, the correct location for the fixation with ID 17 was mapped on the 3D model.

**Figure 16.**
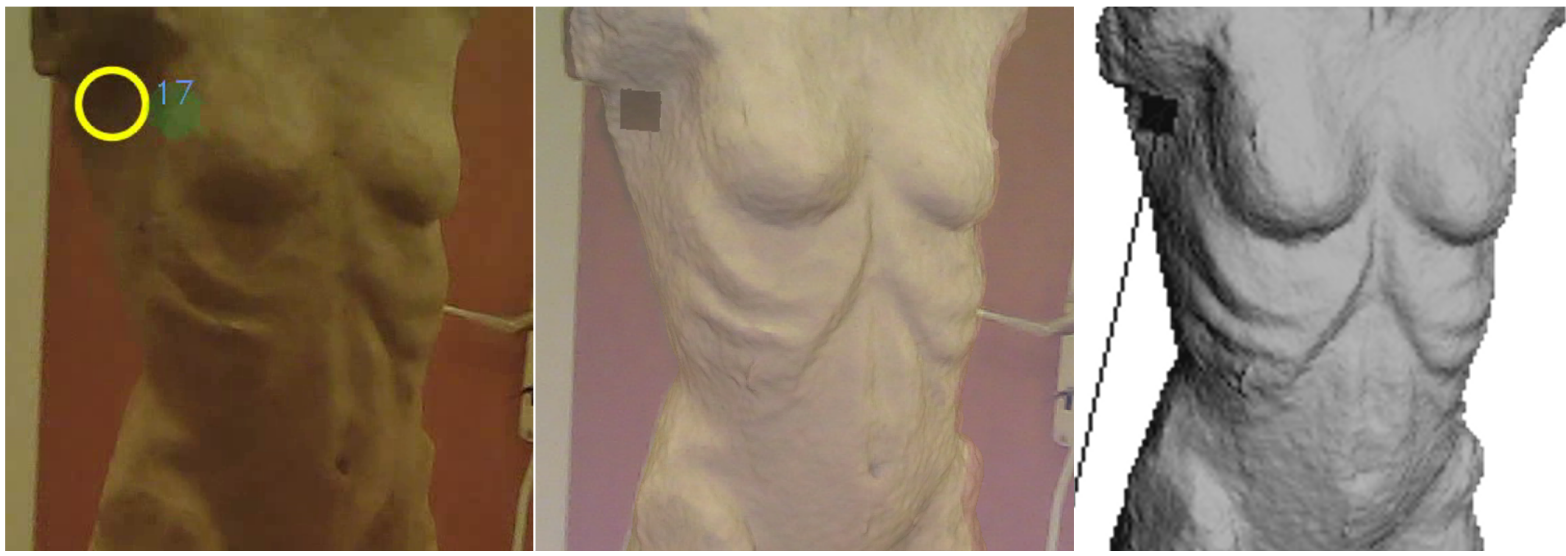
Fixation (ID 17) world_viz_Video (left); View of the camera origin (observer) superimposed semi-transparently over the 3D model in MeshLab and the mapped fixation (ID 17) created with FixationProjector (middle); Fixation (ID 17) on 3D model created with FixationProjector without superimposed camera origin (right).

MeshLab offers the function to assume the position of the observer and to superimpose the image of the camera semi-transparently over the 3D model as can be seen in the middle of Figure 16. The superimposed image clearly shows that the fixation with ID 17 has been mapped to the correct location on the 3D model

Note that the fixation with ID 17 was used as an example for illustration purposes. This test was performed for several fixations whereby comparable results were achieved for all fixations. All fixations tested were correctly registered and mapped in all steps. Therefore, the set criteria for evaluation test I was achieved.

#### Evaluation Test II: Complete MAP3D Procedure

**Figure 17.**
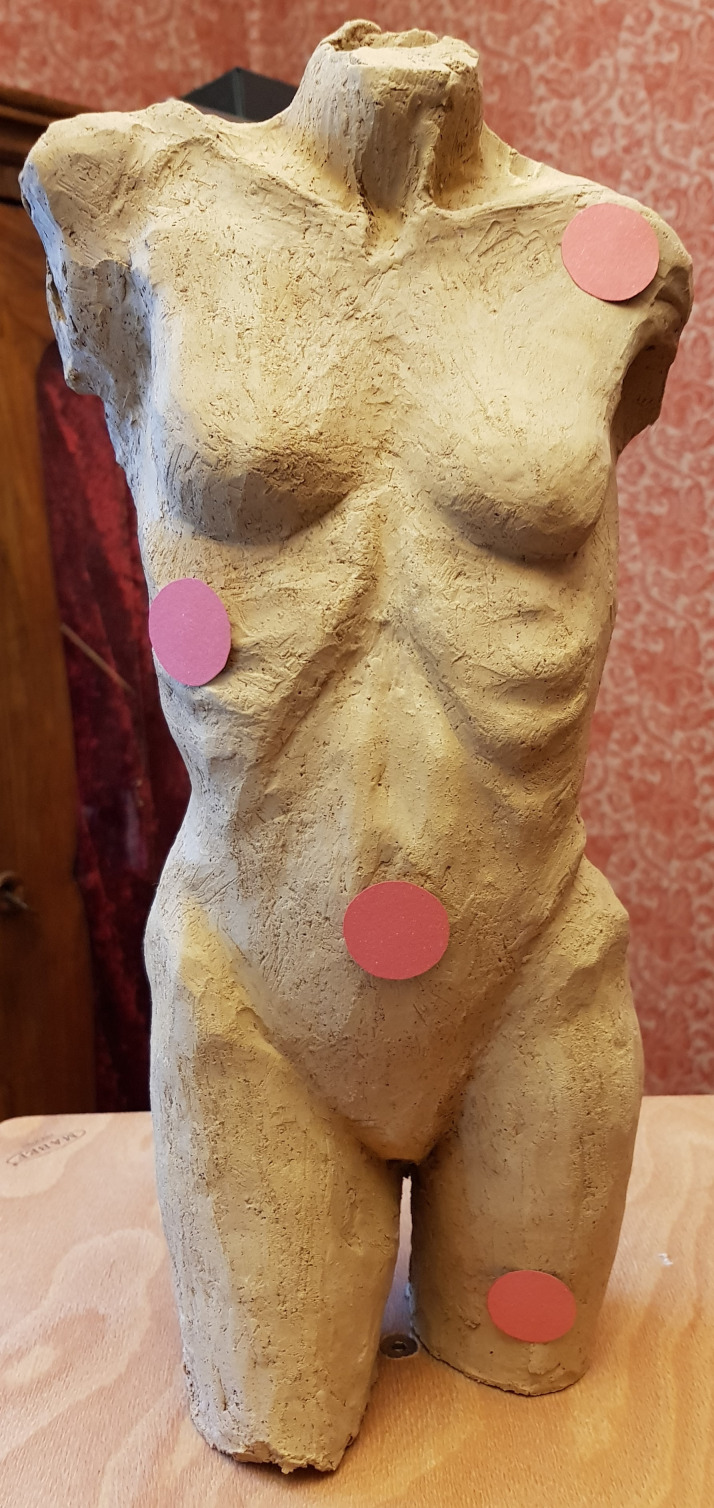
The torso with four red dots.

To evaluate the complete procedure of the MAP3D approach including the eye tracker, an adjusted 3D model was created for exploratory experimentation. For this purpose, four red dots (see Figure 17) Ø 2.5 cm were attached to the stimulus to check the accuracy and especially the subsequent automatic mapping with the FixationProjector.

**Figure 18.**
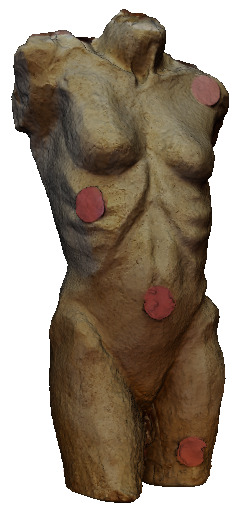
The 3D model of the torso with four red dots.

This newly created 3D model contained these dots (see Figure 18). The participant was wearing the Pupil Labs eye tracker (same eye tracker conditions as in the previous test). The manual marker calibration did not work accurately enough, therefore the so-called “natural feature calibration” was performed. The participant’s task was to look precisely at these dots starting at the top and then moving down step by step. In case the eye tracker is well calibrated and the FixationProjector maps correctly, all dots should be hit accurately. As such the complete procedure of the MAP3D approach can be performed and evaluated.

**Figure 19.**
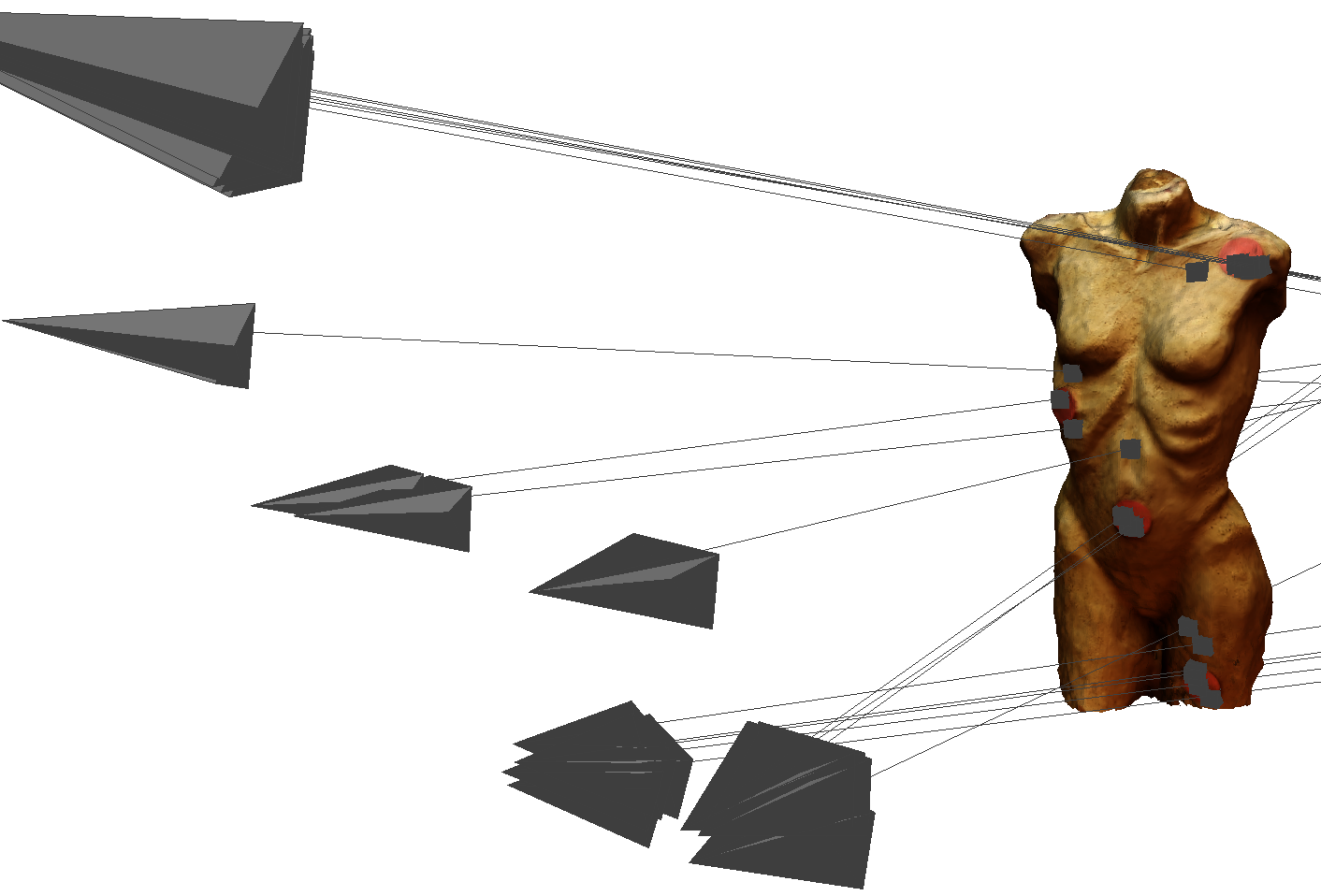
Image of the 3D model with red dots in MeshLab showing the camera origins of the world cam, the visual beams and the 19 mapped fixations.

The Pupil Player software detected 19 fixations during the recording. Following the steps of the MAP3D approach, the .csv output and the MeshLab file, created by FixationProjector, were opened. The .csv. file revealed that all filmstills of the fixations could be matched to the 3D model and that intersection points were calculated for all 19 fixations. At this point, the first criterion (All fixations made are mapped to the 3D model) of evaluation test II was met. The MeshLab output with all 19 fixations set is shown in Figure 19.

**Figure 20.**
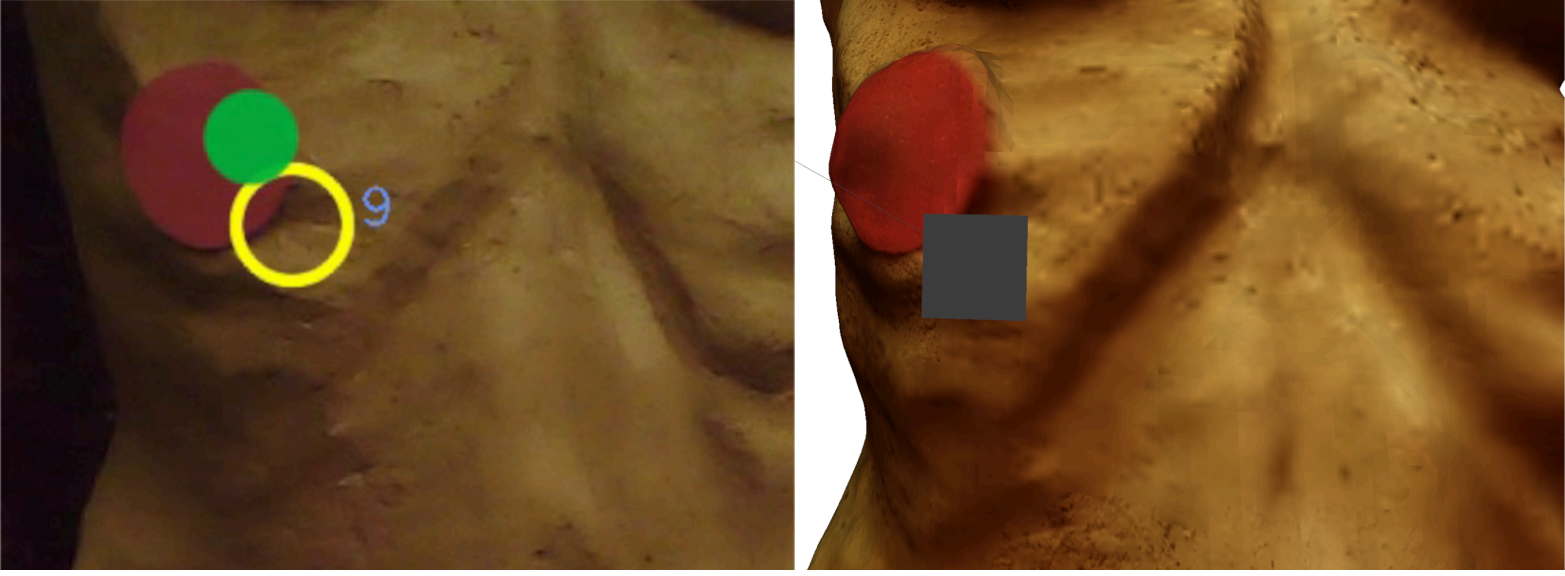
Fixation 9 in the world_viz_video (yellow circle in the left picture) and fixation 9 on the 3D Model with FixationProjector marking (grey square in the right picture).

Next, it was checked whether all red dots were hit. Throughout the recording, a total of 13 fixations were on a red dot, while six fixations were outside of a red dot. This corresponded with the counted number of fixations on a red dot and outside of a red dot in the world_viz_video indicating that all fixations outside the red dots have been recognized as such already by the Pupil Labs software. The FixationProjector has mapped them correctly even if they did not hit the actual target. A detailed example can be seen in Figure 20.

**Figure 21.**
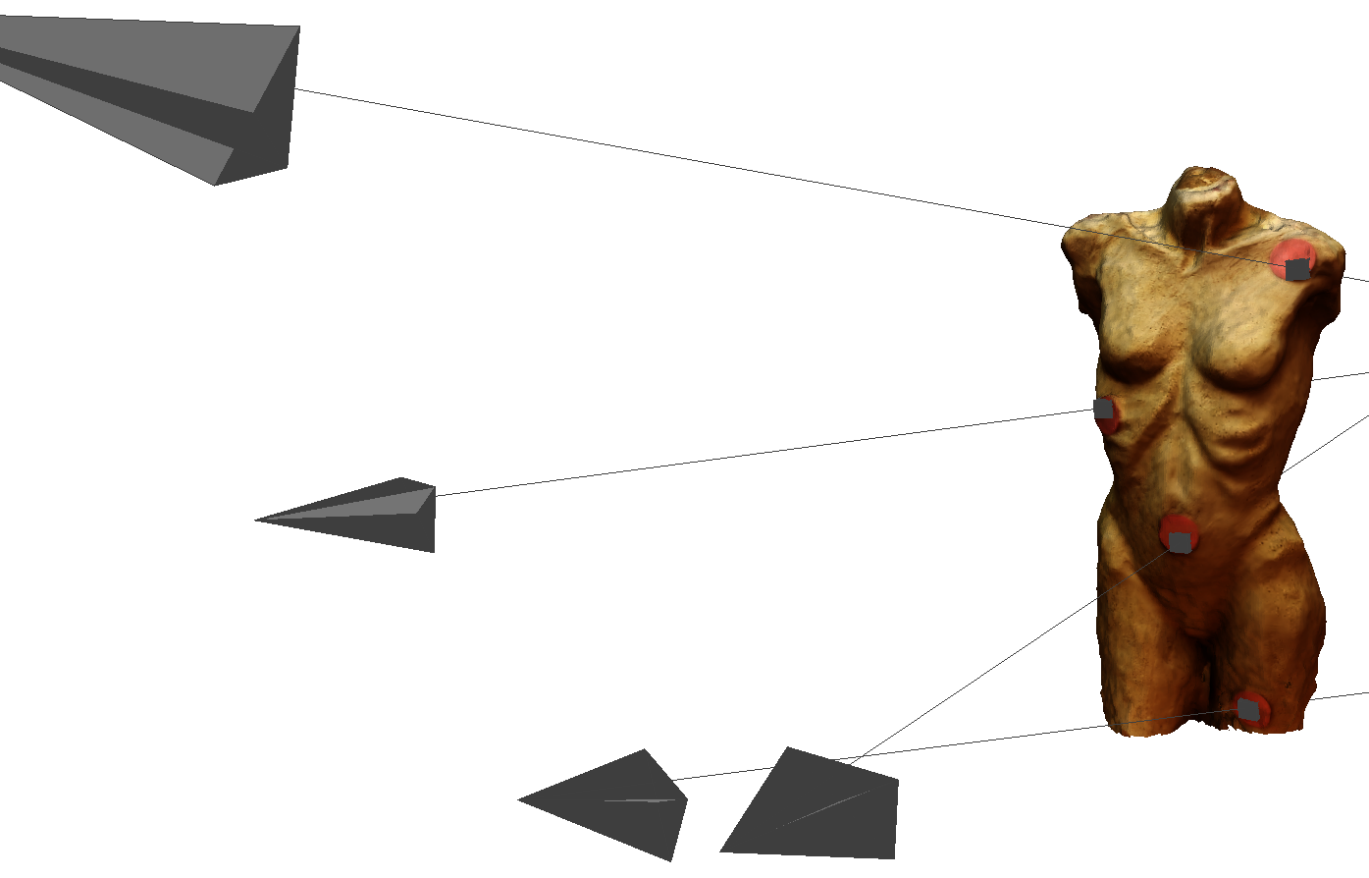
Image of the 3D model with red dots in MeshLab showing one exemplary fixation hitting each dot.

**Figure 22.**
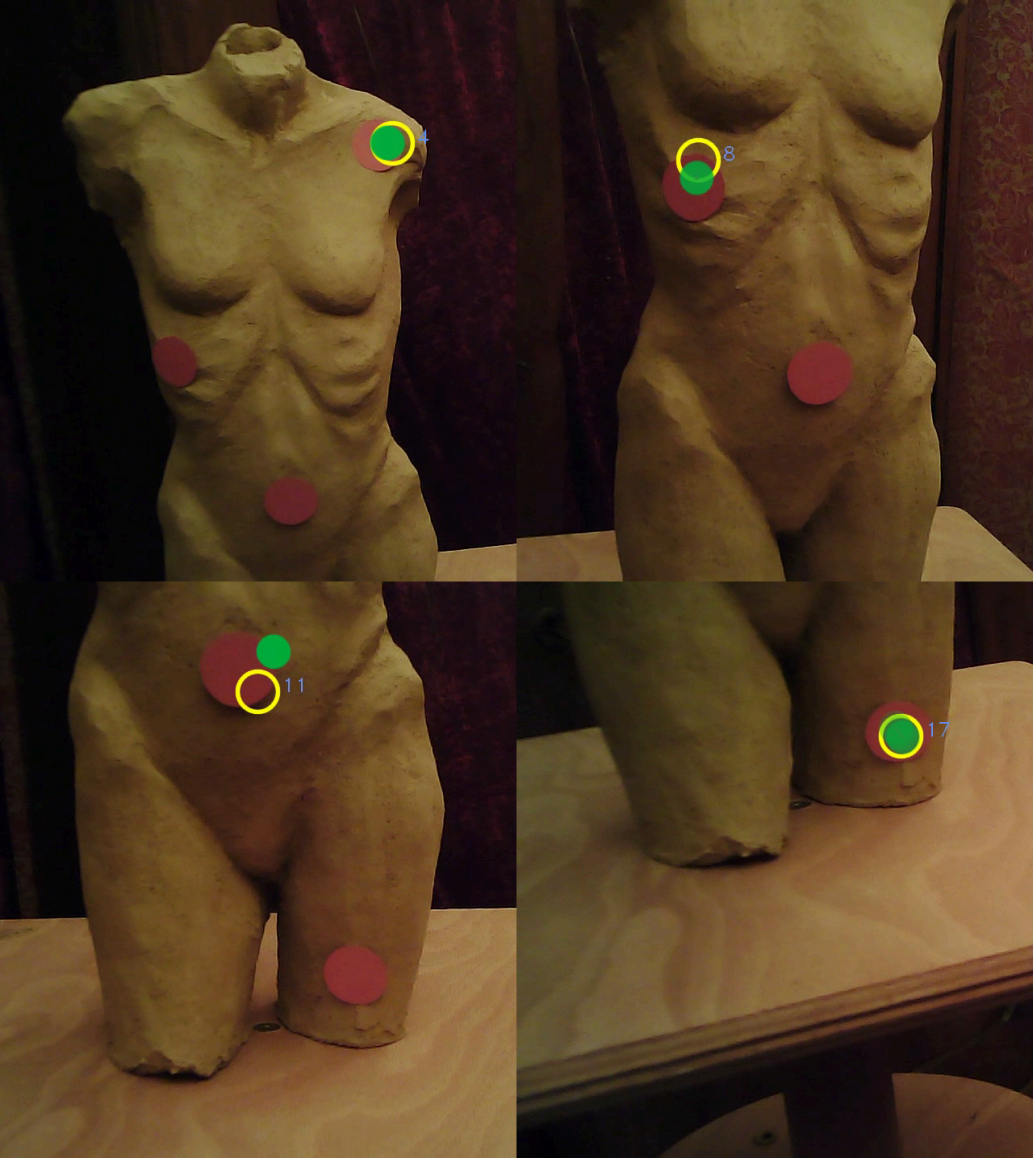
Filmstills of the frames which show the same fixations as in figure 19 extracted from Pupil Labs world_viz_video.

Figure 21 shows an example of each fixation that was mapped onto a red dot (ID 4, 8, 11, 17). Figure 22 shows the corresponding filmstills from the world_viz_video of Pupil Labs with the same fixations.

Overall, it can be stated that all red dots were hit and thus the second criterion of evaluation test II was fulfilled. Thus, fulfilling both criteria of evaluation test II confirms the feasibility and accuracy of the entire eye-tracking process including MAP3D.

#### Evaluation Test III: Comparison between Automatic and Manual Mapping

To evaluate the automatic mapping, 25 participants were asked to manually map the fixations to the reference. Then, their results were compared with the automatic mapping. For this purpose, three filmstills were presented, each from the world_viz_video with fixation (ID 17, ID 49, ID 54) of participant 1 of our application test. A fixation was visualized by Pupil Labs as a yellow circle. The participants had the task to map the three fixations to a reference image (corresponding filmstill from the world video).

**Figure 23.**
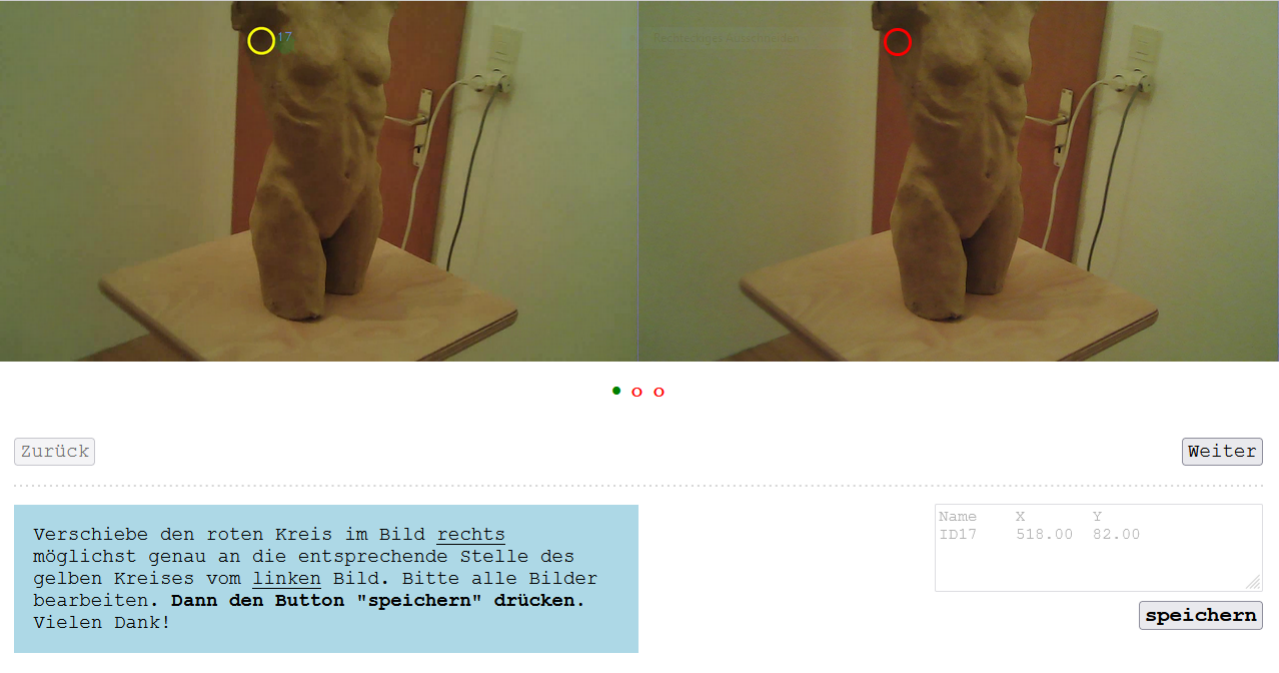
Screenshot of the html tool simulating the manual mapping process.

The manual mapping was carried out in an HTML-based program (see Figure 23). The program collected the x and y coordinates of the respective red circle. Thus, it was possible to compare the accuracy of the manual mapping with the automatic mapping of the MAP3D FrameExtractor. The deviation in pixel from the x and y coordinates of the fixation provided by Pupil Labs was calculated.

**Figure 24.**
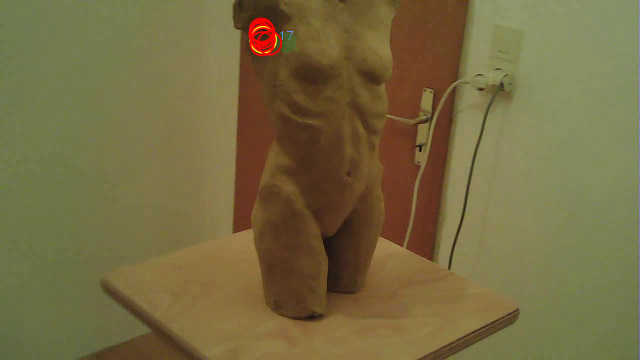
The fixation ID 17 (yellow circle) and the manual mapping of eight participants (red circles) overlapped in one picture.

Figure 24 shows the yellow circle from the world_viz_video and the overlapping mapped red circles by eight of the total 25 participants as an example.

The manual mapping resulted in a mean value of 10.53 (*SD 6.12*), while the MAP3D FrameExtractor had a mean value of 0.53 (*SD 0.23*). Thus, the FrameExtractor led to higher accuracy, so we can conclude that our evaluation criterion is fulfilled with automatic mapping being more accurate than manual mapping.

## Discussion

We developed and explored a photogrammetry-based approach, MAP3D, to generate a virtual 3D model of a stimulus, without the need for complex and expensive scanning tools, technical equipment or markers, and with the opportunity for the participants to move freely in the setting. MAP3D allows mapping the eye movements of one or more participants onto a virtual 3D model of a stimulus by using open-source software. An accurate stimulus representation with a naturally reconstructed surface was created. In addition, rotating the model as well as zooming into relevant areas was possible. The whole viewing process was summarized on one virtual 3D model and in one common 3D coordinate system; x, y and z coordinates of the fixations were available in a .csv file for analysis.

Evaluating MAP3D revealed that the fixations were mapped to the correct positions on the 3D model, the predefined targets were hit, and all fixations were automatically mapped onto the 3D model. MAP3D facilitated the analysis of eye-tracking data provided by a mobile eye tracker and the automatic mapping appeared more user-friendly than the manual or semi-manual mapping process with other solutions.

### Advantages

Due to the chosen technique of photogrammetry for the MAP3D approach, we see several advantages in connection with eye-tracking research. First, it is possible to map the whole gaze points, not only fixations as in our exploration, onto the 3D model with MAP3D if researchers are interested in analyzing the complete process. For this step, the x and y coordinates of the gaze points need to be determined by the eye-tracking software in a .csv file. Second, MAP3D can analyze several stimuli of interest located in a room by constructing a 3D model of the whole environment. Third, the 3D models can be created from the videos of eye-tracking recordings, so that it is possible to analyze recordings that were previously recorded and had not yet taken 3D aspects into account. Fourth, by reconstructing a 3D model with photogrammetry, the origins of the cameras are also traceable identifying participants’ position and viewing perspective. Thus, additional data is collected and no further equipment like e.g. magnetic trackers is required.

With MAP3D we follow a fixation-based approach. As such, a complete fixation sequence can be mapped onto one 3D model without being distorted or incomplete, which would be the case when evaluated over multiple 2D reference images (25). Moreover, the degree of accuracy of the 3D model and realistic rendering is higher than in other 3D eye-tracking approaches that work for example with virtual cubed volumes of interest (VOI) around the stimulus (19, 20). However, whether this detailed reproduction and the effort for creating the model is necessary, depends of course on the research field and research question. In art reception, particularly in the domain of sculpting, a reproduction as accurate as possible is desirable. Yet, this pays its fee in terms of efficiency as the creation of the reference model can take some time depending on the degree of perfection. Regardless of the perfection, there are several challenges and limitations for the automatic mapping and the creation of the 3D model of a stimulus with photogrammetry.

### Limitations, Challenges, and Possible Solutions

In cases where the camera quality was poor or the lighting conditions differed during recording the eye movements and the reference views for the 3D model, it happened that some fixations, which were recognized by the Pupil Labs software, were not automatically transferred to the 3D model. Thus, the filmstills could not be assigned to the model by photogrammetry due to a lack of matches. However, as those cases were documented in the .csv file created by the FixationProjector tool, it was possible to manually map these missing fixations to the 3D model. In our third evaluation, we paid more attention to uniform lighting conditions and the reference images were taken in landscape format to match the world camera resulting in an improved matching in VisualSfM with all fixations being automatically mapped to the 3D model with the FixationProjector tool.

Kollert et al. (12) also noted in their study that the pure use of eye-tracking frames can be insufficient for the creation of a 3D model. Therefore, they used an additional LiDAR scanner, further images, and they incorporated manual refinements to achieve their intended result. Similarly, our exploration also showed that the fidelity and completeness of the 3D model improved by adding pictures with high quality and different angles. Using the frames of the eye tracker video only resulted in insufficient quality due to the low resolution of the world camera. To avoid this problem, the eye tracker could be equipped with a higher resolution world camera. Moreover, researchers are advised to make a “reference recording” first instead of using the actual recording of the participants as a reference. To create the densest possible point cloud for the 3D model, the “reference recording” is done by circling the stimulus 360° slowly with the mobile eye tracker at least at two different heights. When moving too quickly, motion blur may occur. Bici et al. (1) propose a very similar approach regarding their reference images for the 3D model of a statue taken with a camera on a tripod. They used four different heights and call this approach a "cylindrical virtual cage"(20, p. 4).

During our application test, it was also noticed that it is difficult to define a rule of thumb for the number of images that should be used for the reconstruction of the stimulus. This is in line with Westoby et al. (30), who stressed that it is impossible to provide a guideline for the number of images needed for the most accurate reconstruction due to the different textures, lighting conditions and materials in different scenes. Therefore, researchers should try to ensure constant or similar lighting conditions between recordings.

Some eye trackers work with a wide-range camera, which often comes along with a fish eye effect that can cause distortions in the resulting 3D model. The Pupil Labs eye tracker that was used in our application test, has two different lenses for the 120 Hz camera. A wide-angle lens and a narrow-angle lens. The wide-angle lens led to severe distortions when creating the 3D model. Therefore, the test was performed with the narrow-angle lens, which is suboptimal as the FOV of the world camera is limited and can affect the matching using photogrammetry negatively. However, there are methods available to handle radial distortion problems in photogrammetry (17).

Another limitation lies in the technique of photogrammetry itself, which is relevant for the selection of stimulus material. In the case of objects with highly reflecting surfaces, the reconstruction works poorly (29). In these cases, it is possible to apply a completely removable spray to the objects to make them appear mat. Alternatively, Bici et al. (1) have developed a workflow to deal with reflective surfaces in photogrammetry without using such drastic methods as matting sprays, which can be a problem when dealing with delicate stimuli such as artwork. They show how the problem can be solved from the software side, by reconstructing a bronze statue. Other options can be approaches based on creating a 3D model with the help of a scanner or an RGB-D camera like Microsoft Kinect as suggested by Pfeiffer et al. (20) or Paletta et al. (16).

Moving objects in the setting are also challenging for photogrammetry and thus also for the MAP3D approach. Pfeiffer et al. (20) have dealt with dynamic scenes in their marker and Microsoft Kinect-based approach. Moving objects were not the focus of our exploration, but interested readers are referred to Schöning et al (22), who offer solutions for photogrammetry-based approaches. They created accurate 3D models out of video data including moving objects.

MAP3D in its current state is a post-hoc application. Depending on the research question, a real-time method, such as the marker-based method of Pfeiffer et al. (19, 20) may be more suitable for data collection.

### Future Directions

MAP3D is an explorative approach for the automatic mapping of real world eye-tracking data on a virtual 3D model that we aim to develop and improve further. Several future directions are outlined here to illustrate the possibilities for eye-tracking research.

In future, it will be possible to visualize the fixation data on the 3D model as a scanpath. A fixation will be represented by a sphere similar to the circles known from 2D scanpaths. The size of the sphere will depend on the fixation duration. Currently, we are working on a solution to automatize this visualization. It is also planned to add VOIs as well as gridded VOIs (cubes) to the MAP3D approach allowing different types of quantitative analysis.

Furthermore, saliency maps like heat maps, attention maps and other point-based methods will be added. For example, the modified versions of the Kulback-Leibler-divergence and the ROC analysis from Singh et al. (23) are conceivable methods for attention map-based, quantitative analyses. Singh et al (23) adapted these methods to meshed 3D models on a per-triangle basis rather than a per-pixel basis. Pfeiffer and Memilis’s (18) approach for generating realistic 3D heat maps supports binocular perspectives and depth of focus. These important aspects in spatial perception can be considered in future heat map development for the MAP3D approach.

Since the event detection is not done by MAP3D, but by the respective eye-tracking software, it is important to note that MAP3D uses only one vector for the mapping. Therefore, the correction of the parallax error by the eye-tracking software is used. As an extension, one can consider the vergence and include two vectors or rays of vision in the analysis. Moreover, the use of two scene cameras instead of the standard single camera on the eye tracker could be helpful here to increase the accuracy of the 3D model as well as the 3D mapping. Interesting approaches can also result from analyzing the viewpoints of the observers. For example, the path of the viewer could be simultaneously analyzed with the scanpath.

MAP3D is not restricted to one eye tracker brand like other 3D analysis solutions are. It can be easily adapted to use the data from various eye trackers in the future. This requires small changes in the source code to be able to process the data of the corresponding eye tracker correctly. After all, each company designs the output files somewhat differently and the naming is not consistent either.

To make the application even simpler we have designed a simple graphical user interface. Currently, MAP3D is to be understood as a prototype, which needs further exploration and can continue to grow with the help of the open-source community. The developed command line programs and the graphical user interface will be open to the public via GitHub. It is a free available low-cost, open-source solution (Link available after publication).

### Ethics and Conflict of Interest

The authors declare that the contents of the article are in agreement with the ethics described in http://biblio.unibe.ch/portale/elibrary/BOP/jemr/ethics.html and that there is no conflict of interest regarding the publication of this paper.

### Acknowledgements

We are grateful to all persons who provided valuable feedback on previous drafts of the paper, supported us with technical advice, and participated in the exploratory experimentation.
